# Diaphragmatic ultrasound can help evaluate pulmonary dysfunction in patients with stroke

**DOI:** 10.3389/fneur.2023.1061003

**Published:** 2023-04-18

**Authors:** Yifei Chen, Shuyan Zhou, Lixia Liao, Jinyan He, Danzhe Tang, Wen Wu, Kangling Wang

**Affiliations:** Department of Rehabilitation Medicine, Zhujiang Hospital, Southern Medical University, Guangzhou, China

**Keywords:** stroke, pulmonary function, diaphragmatic ultrasound, evaluation, motor function

## Abstract

**Objective:**

Pulmonary dysfunction after stroke is increasingly gaining attention from clinical and rehabilitation specialists. However, owing to cognitive and motor dysfunction in patients with stroke, determining the pulmonary function of these patients remains challenging. The present study aimed to devise a simple method for an early evaluation of pulmonary dysfunction in patients with stroke.

**Methods:**

Overall, 41 patients with stroke in the recovery period (stroke group) and 22 matched healthy controls (control group) were included in the study. We first collected data regarding baseline characteristics for all participants. Furthermore, the participants with stroke were examined using additional scales, such as the National Institutes of Health Stroke Scale (NIHSS), Fugl–Meyer assessment scale (FMA), and modified Barthel Index (MBI). Subsequently, we examined the participants with simple pulmonary function detection and diaphragm ultrasound (B-mode). Ultrasound indices calculated were as follows: the thickness of the diaphragm under the position of functional residual capacity (TdiFRC), the thickness of the diaphragm under the position of forced vital capacity (TdiFVC), thickness fraction, and diaphragmatic mobility. Finally, we compared and analyzed all data to identify group differences, the correlation between pulmonary function and diaphragmatic ultrasound indices, and the correlation between pulmonary function and assessment scale scores in patients with stroke, respectively.

**Results:**

Compared with the control group, patients in the stroke group exhibited lower values for indices of pulmonary and diaphragmatic function (*p* < 0.001), except for TdiFRC (*p* > 0.05). The majority of the patients with stroke had restrictive ventilatory dysfunction, as indicated by a significantly higher incidence ratio (36 in 41 patients) than that in the control group (0 in 22 patients) (*p* < 0.001). Moreover, significant correlations were found between pulmonary function and diaphragmatic ultrasound indices (*p* < 0.05), with the strongest correlation between TdiFVC and pulmonary indices. In the stroke group, pulmonary function indices were negatively correlated with the NIHSS scores (*p* < 0.001) and positively correlated with the FMA scores (*p* < 0.001). No (*p* > 0.05) or weak (*p* < 0.05) correlation was found between pulmonary function indices and the MBI scores.

**Conclusion:**

We found that patients with stroke had pulmonary dysfunction even in the recovery period. Diaphragmatic ultrasound can be used as a simple and effective tool for detecting pulmonary dysfunction in patients with stroke, with TdiFVC being the most effective index.

## Introduction

Stroke is the second leading cause of death and the third leading cause of disability worldwide (1). Patients who have suffered a stroke frequently complain of motor and speech disorders as well as dysphagia. However, pulmonary dysfunction after stroke is gradually receiving increased attention. Pulmonary dysfunction can lead to a decline in cardiopulmonary function and activity endurance while simultaneously increasing the risk of pulmonary complications, thereby affecting patients’ motor and speech recovery and their quality of life (2). Therefore, the prompt detection of pulmonary dysfunction among patients with stroke will be of great significance to perform respiratory rehabilitation correspondingly.

Although there are several established methods for assessing respiratory function (3), assessment in patients with stroke is often limited owing to deficits in cognitive, speech, or motor domains. To date, simple pulmonary function assessment has been recognized as one of the most commonly used methods. Schermer et al. (4) confirmed that the indicators of simple respiratory function assessment have remarkable reliability and validity. However, this method also requires patients with stroke to have sufficient cognitive abilities to understand the basics of the examination, which even a healthy older adult may find difficult to understand, and it requires them to be able to cooperate throughout the examination. Therefore, it is important to find a convenient and convincing way for the assessment.

Diaphragmatic ultrasound is a commonly used method for measuring diaphragmatic activity. Previous studies have reported a certain relationship between diaphragmatic ultrasound indices and pulmonary function parameters in some diseases, including osteoporosis, vertebral fracture, kyphosis, late-onset Pompe disease, and amyotrophic lateral sclerosis (5–7). To the best of our knowledge, only two studies have reported the correlation between pulmonary function indices and diaphragmatic ultrasound in patients with stroke to date. In one study, Jung et al. (8) reported a statistically significant correlation between diaphragmatic thickness (DT), thickness ratio, and diaphragmatic excursion and between forced vital capacity (FVC), forced expiratory volume in 1 s (FEV1), and peak expiratory flow in patients with stroke compared to healthy controls. In another study, Kim et al. (9) reported a positive correlation between the thickening ratio (%), the ratio of diaphragm thickness at end expiration to the thickness at total lung capacity (TCL), and maximal inspiratory pressure (MIP). However, their study measured the ultrasound index for only one diaphragm, and the correlation with the pulmonary function was restricted to only inspiratory muscle function. Thus, further studies are required to identify the relationship between diaphragmatic ultrasound and pulmonary function.

In the present study, we assessed and compared the pulmonary and diaphragm function of patients with stroke by combining a simple respiratory function assessment with diaphragmatic ultrasound detection. We hypothesized that there is a correlation between diaphragmatic ultrasonography and pulmonary function in patients with stroke. Because ultrasound offers the advantage of visually observing morphologic changes in muscle function (10), a convincing correlation with the pulmonary function will be of great help and convenience to physicians in the early detection of pulmonary dysfunction for patients with stroke.

## Materials and methods

### Participants

The sample size of the present study was determined based on the sample sizes of previous similar studies (11, 12). Overall, 51 patients with hemiplegia in the convalescent stage of stroke (stroke group) and 22 age-and gender-matched healthy volunteers (control group) were enrolled.

The inclusion criteria for the stroke group were as follows: (1) patients diagnosed with stroke in accordance with the diagnostic criteria by the Chinese classification of cerebrovascular diseases (2015) and confirmed by brain computed tomography or magnetic resonance imaging findings, (2) those aged 18–70 years, (3) those diagnosed with stroke for the first time, with a course of stroke ranging from 1 month to 1 year, (4) those with right hemiplegia, (5) those who were conscious and had stable vital signs with no progression of neurological symptoms, (6) those who had normal cognitive function (mini-mental state examination [MMSE] score > 26) and were able to complete the entire examination, and (7) those who voluntarily participated and provided written informed consent. The exclusion criteria were as follows: (1) patients presenting with a previous history of respiratory diseases, including chronic bronchopneumonia, chronic obstructive pulmonary disease, bronchiectasis, and lung cancer, (2) those with severe skeletal deformities of the thorax or spine, (3) those with neuromuscular junction disease, (4) those with tracheotomy or auxiliary ventilation, (5) those presenting with a history of thoracic or abdominal surgery, (6) those presenting with a history of receiving respiratory training, (7) those presenting with a history of using inhaled corticosteroids or respiratory central stimulants, (8) those whose House–Brackmann assessment revealed facial paralysis of >grade II, (9) those with severe cardiac, lung, kidney, liver, or other organ dysfunctions, (10) those with severe cognitive impairment or mental illness causing inability in cooperation, and (11) pregnant or lactating women.

The inclusion criteria for the control group were as follows: (1) individuals with no previous history of cerebrovascular disease, (2) those aged between 18 and 70 years, and (3) those who were conscious and had stable vital signs and normal cognitive function (MMSE score > 26 points), were cooperative, and who voluntarily participated and signed the informed consent form. The exclusion criteria for the control group were the same as those for the stroke group.

The following were the rejection and shedding criteria: (1) individuals who did not undergo qualified pulmonary function index measurements and (2) those whose ultrasound examination did not reveal clear diaphragmatic images.

### Research design

This was a prospective cross-sectional study that aimed to assess patients with stroke who were admitted to a rehabilitation unit of Zhujiang Hospital. Moreover, healthy controls were recruited *via* poster advertisements on local notice boards.

### Data acquisition

Basic information, including age, gender, height, weight, and smoking history, were collected for all participants. Additional clinical information, including the course of the disease, type of stroke, and brain lesion location, was collected for the patients in the stroke group.

Pulmonary function index values, including FVC, FEV1, MIP, and maximum expiratory pressure (MEP), were collected using a portable pulmonary function detector (Sike x1; Xiamen Saike Medical Equipment Co., Ltd.).

Diaphragmatic function index values, including the thickness of the diaphragm under the position of functional residual capacity (TdiFRC), the maximum thickness of the diaphragm under the position of forced vital capacity (TdiFVC), and diaphragmatic motion (DM) of the right side of the diaphragm, were collected using full digital color Doppler ultrasound (F8000; Shenzhen Dewell Medical Electronics Co., Ltd.).

#### DT measurement

All participants were placed in the supine position. To observe the region between ribs 8 and 9 and to obtain two-dimensional images, the chest wall was perpendicularly illuminated using a linear transducer. With the ultrasound probe perpendicular to the two ribs, the diaphragm was visualized as a hypoechoic area between the two hyperechoic layers (i.e., diaphragmatic pleura and peritoneum), and DT was measured from this area at the end of inspiration and expiration. The mean value of three measurements was used for the analysis (3).

The thickness fraction (TF) of the diaphragm was calculated using the following formula:

TF = (maximum DT at the end of deep inspiration − DT at the end of quiet expiration)/DT at the end of quiet expiration × 100% (13–15).

#### Diaphragm motion

Diaphragm motion was assessed using ultrasound in M-mode. As described in previous studies (11, 16, 17), the amplitudes were measured with one caliper placed at the baseline of the diaphragm echoic line and the other placed at the apex or the maximum height of the line. At least three measurements were recorded, and the average value of these measurements was used for statistical analysis.

All patients in the stroke group were examined using the National Institutes of Health Stroke Scale (NIHSS), Fugl–Meyer assessment scale (FMA), and modified Barthel Index (MBI) to evaluate their severity of brain damage, motor ability, and activities of daily living (ADL), respectively.

### Statistical analysis

All statistical analyses were performed using Statistical Product and Service Solution version 25.0 statistical software (IBM Corporation, Armonk, NY, United States). The Shapiro–Wilk tests were used to examine normal distribution. The two-sample *t*-test and nonparametric Mann–Whitney tests were used to compare the two groups in terms of continuous variables, whereas the *χ*^2^ test was used for comparison in terms of categorical variables. Pearson correlations were used to measure the relationship between pulmonary and diaphragmatic ultrasound indices and between pulmonary indices and NIHSS, FMA, and MBI scores, respectively. Measurement data were expressed as mean (standard deviation), whereas counting data were expressed as frequencies. The level of statistical significance was set at a *p*-value of <0.05.

## Results

### Basic information

**Figure 1:**
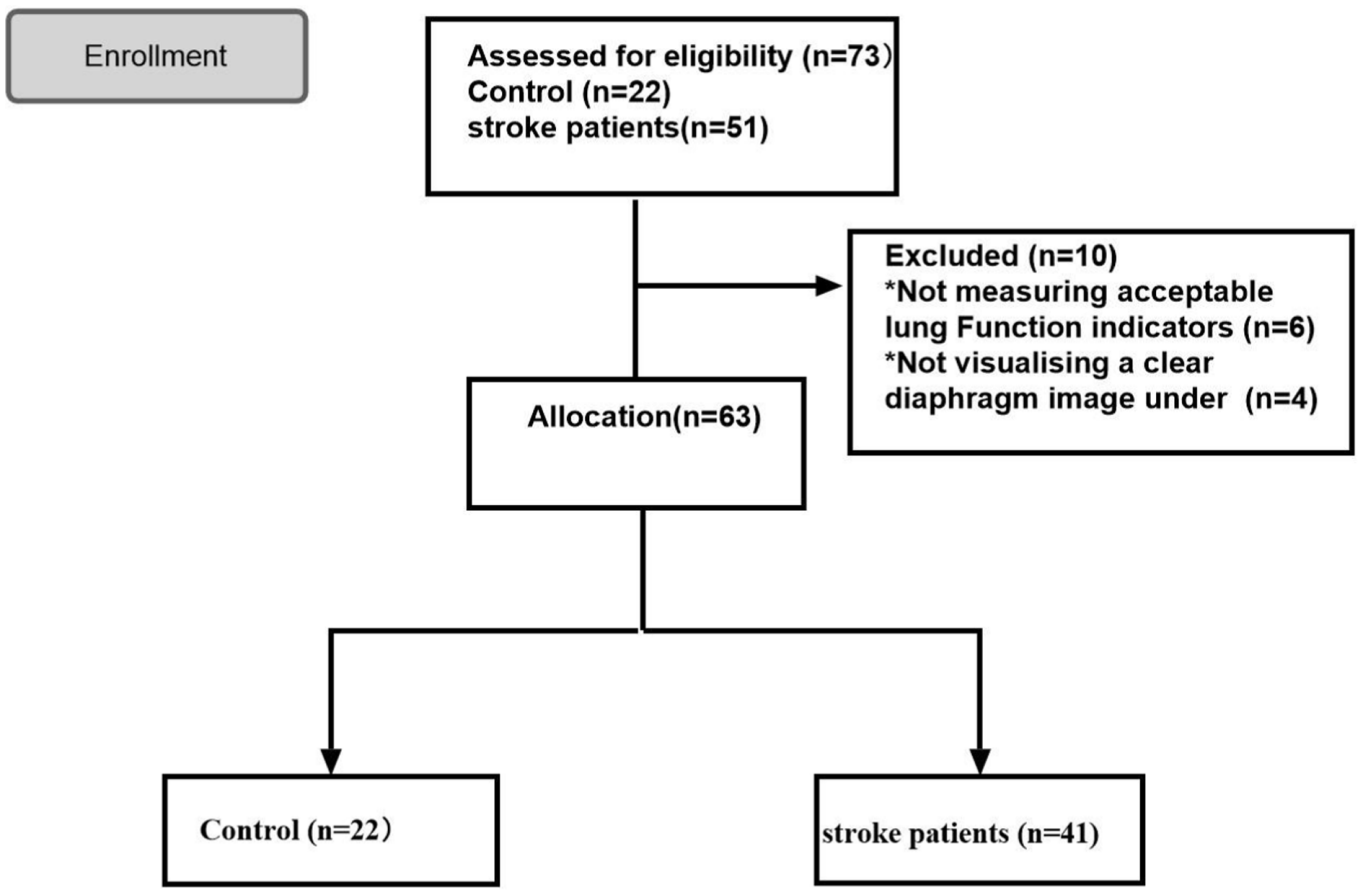
Trial flowchart.

Overall, 73 participants were included in this study and classified into the stroke (*n* = 51) and control (*n* = 22) groups. Of the patients in the stroke group, six and four patients were excluded as their qualified pulmonary function index was not measured and a clear diaphragmatic image was not visualized under ultrasound, respectively. Finally, 63 participants were enrolled in the study, including 41 patients with stroke and 22 controls (Figure 1).

No significant differences were identified between the two groups in terms of basic information (*p* > 0.05). However, compared with individuals in the control group, patients in the stroke group demonstrated significantly lower values for all pulmonary indices (*p* < 0.001) and most diaphragmatic indices (*p* < 0.05), except for TdiFRC (*p* = 0.404). Notably, in the stroke group, 36 patients were diagnosed with restrictive ventilatory disorder. The incidence of restrictive ventilatory disorder in these patients was higher than that in the control group (0 in 22 patients, *p* < 0.001) (Table 1).

**Table 1:** Baseline characteristics of all participants (*n* = 63).

	Stroke group (*n* = 41)	Control group (*n* = 22)	*t*/χ^2^	*p*
*Characteristic*				
Sex (male/female)	26/15	12/10	0.471	0.493
Age (years)	55.61 ± 12.37	50.05 ± 10.92	−1.771	0.082
Height (cm)	1.66 ± 0.07	1.64 ± 0.08	−0.801	0.462
Weight (kg)	63.80 ± 10.46	63.77 ± 9.73	−0.12	0.991
BMI	23.04 ± 2.89	23.55 ± 2.88	0.679	0.500
Smoking history (yes/no)	12/29	5/17	0.492	0.580
Stroke type (hemorrhage/infarction)	19/22	–	–	–
Course of disease (days)	85.8 ± 62.8	–	–	–
NIHSS score	9.4 ± 5.2	–	–	–
FMA score	42.00 ± 22.80	–	–	–
MBI score	53.29 ± 24.00	–	–	–
*Lung function index*				
FVC (L)	2.00 ± 0.52	3.53 ± 0.81	7.955	**<0.001**
FEV1 (L)	1.58 ± 0.44	2.88 ± 0.74	7.517	**<0.001**
MIP (cmH2O)	35.95 ± 15.12	93.14 ± 12.69	13.479	**<0.001**
MEP (cmH2O)	49.41 ± 15.95	131.91 ± 38.73	9.564	**<0.001**
*Diaphragmatic ultrasound indicators*				
TdiFRC (cm)	0.20 ± 0.02	0.21 ± 0.02	1.010	0.404
TdiFVC (cm)	0.36 ± 0.05	0.44 ± 0.06	5.530	**<0.001**
TF (%)	85.51 ± 21.06	110.38 ± 17.46	4.730	**<0.001**
DM (cm)	1.76 ± 0.24	1.93 ± 0.29	2.553	**0.013**
*Types of ventilatory disorders*				
Restrictive	36/41	0/22	37.062	**<0.001**
Obstructive	1/41	1/22	–	–
Mixed	2/41	0/22	–	–

NIHSS, National Institutes of Health Stroke; FMA, Fugl–Meyer Assessment; MBI, Modified Barthel Index; FVC, functional vital capacity; FEV, forced expiratory volume in 1 s; MIP, maximum inspiratory pressure; MEP, maximum expiratory pressure; TdiFRC, Thickness of Diaphragm under Position of Functional Residual Capacity; TdiFVC, Thickness of Diaphragm under Position of Forced Vital Capacity; TF, thickening fraction; DM, diaphragmatic mobility. Bold indicates statistical significance.

### Correlations between pulmonary function and diaphragmatic indices

**Figure 2:**
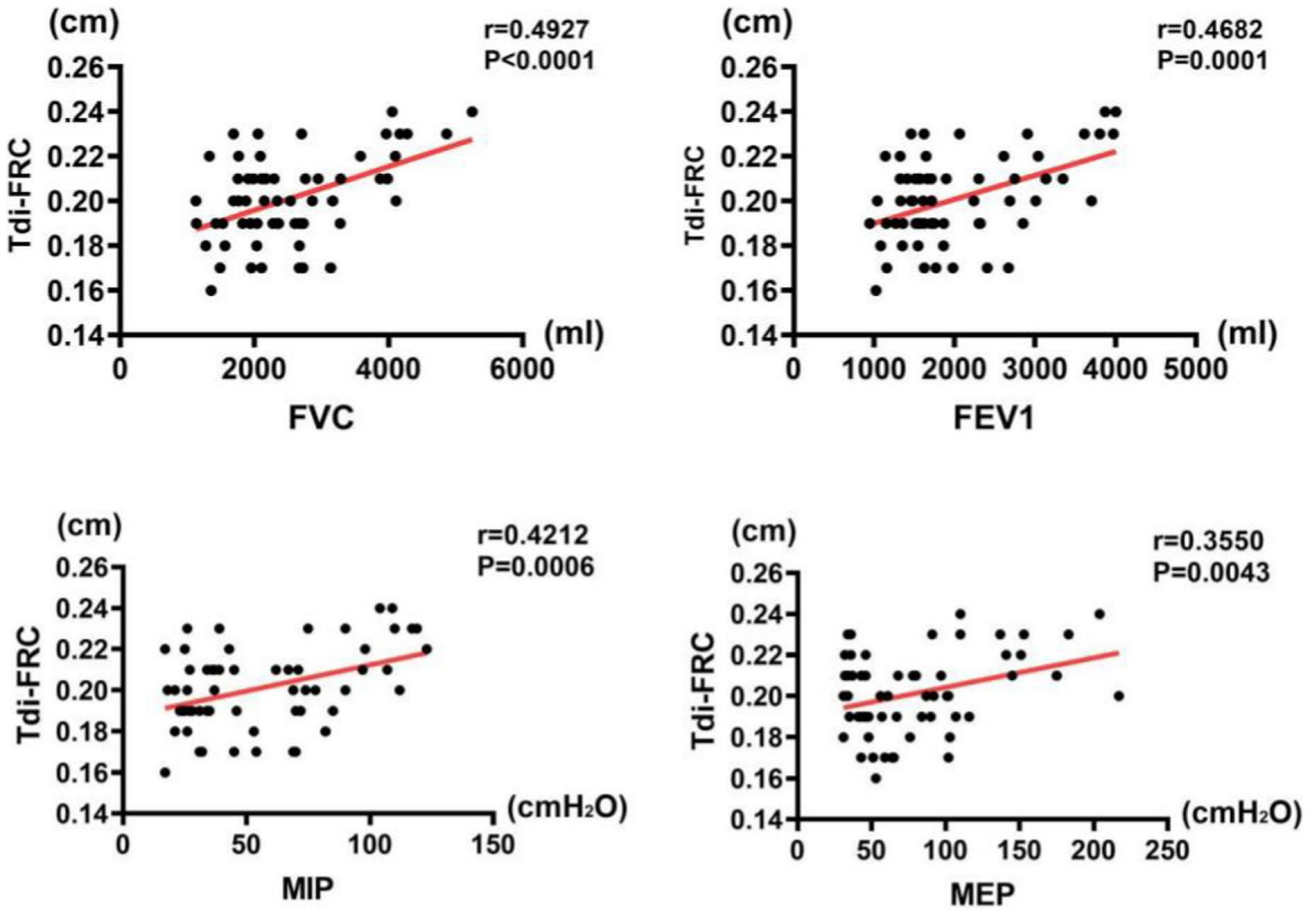
Correlations between pulmonary function indices and TdiFRC.

**Figure 3:**
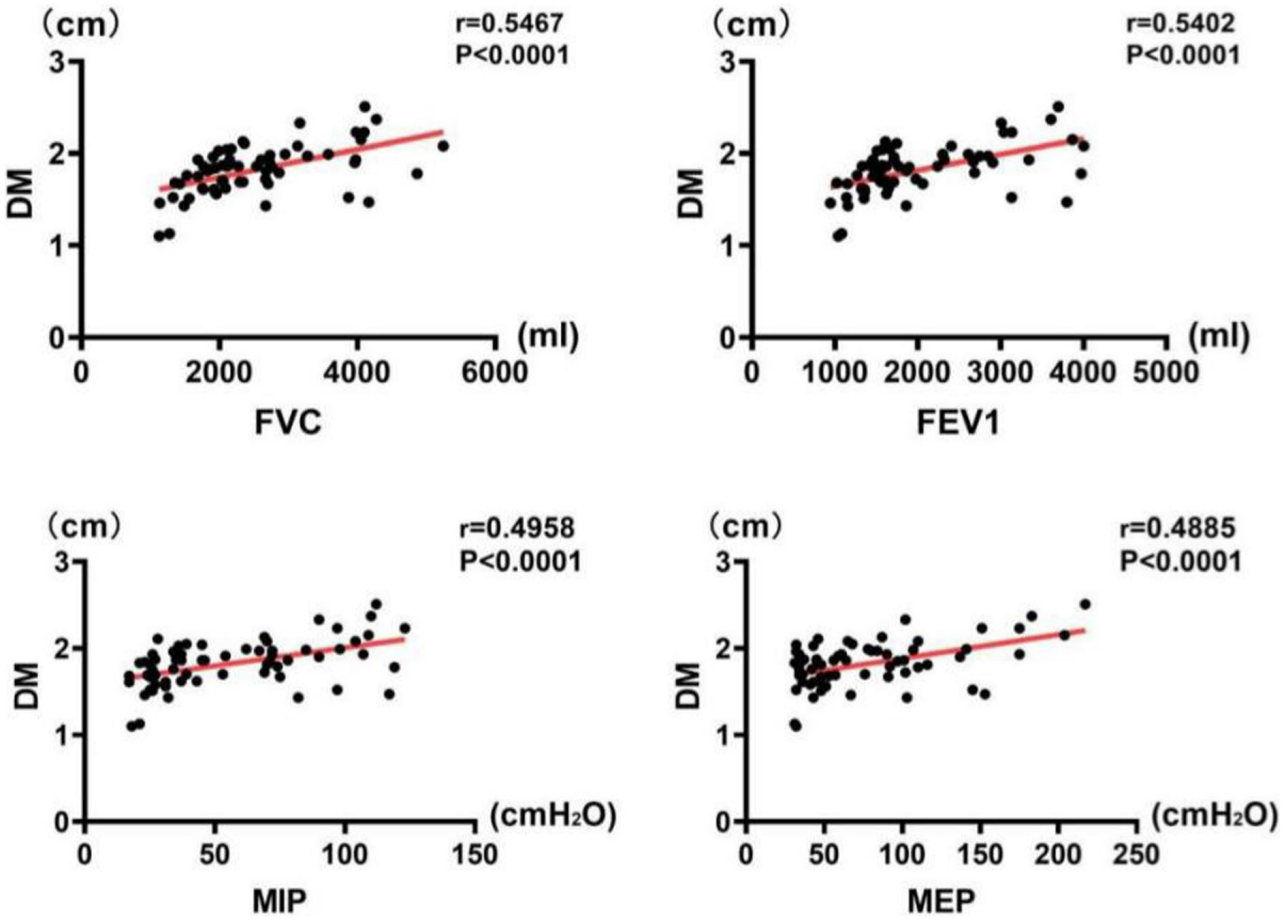
Correlations between pulmonary function indices and DM.

**Figure 4:**
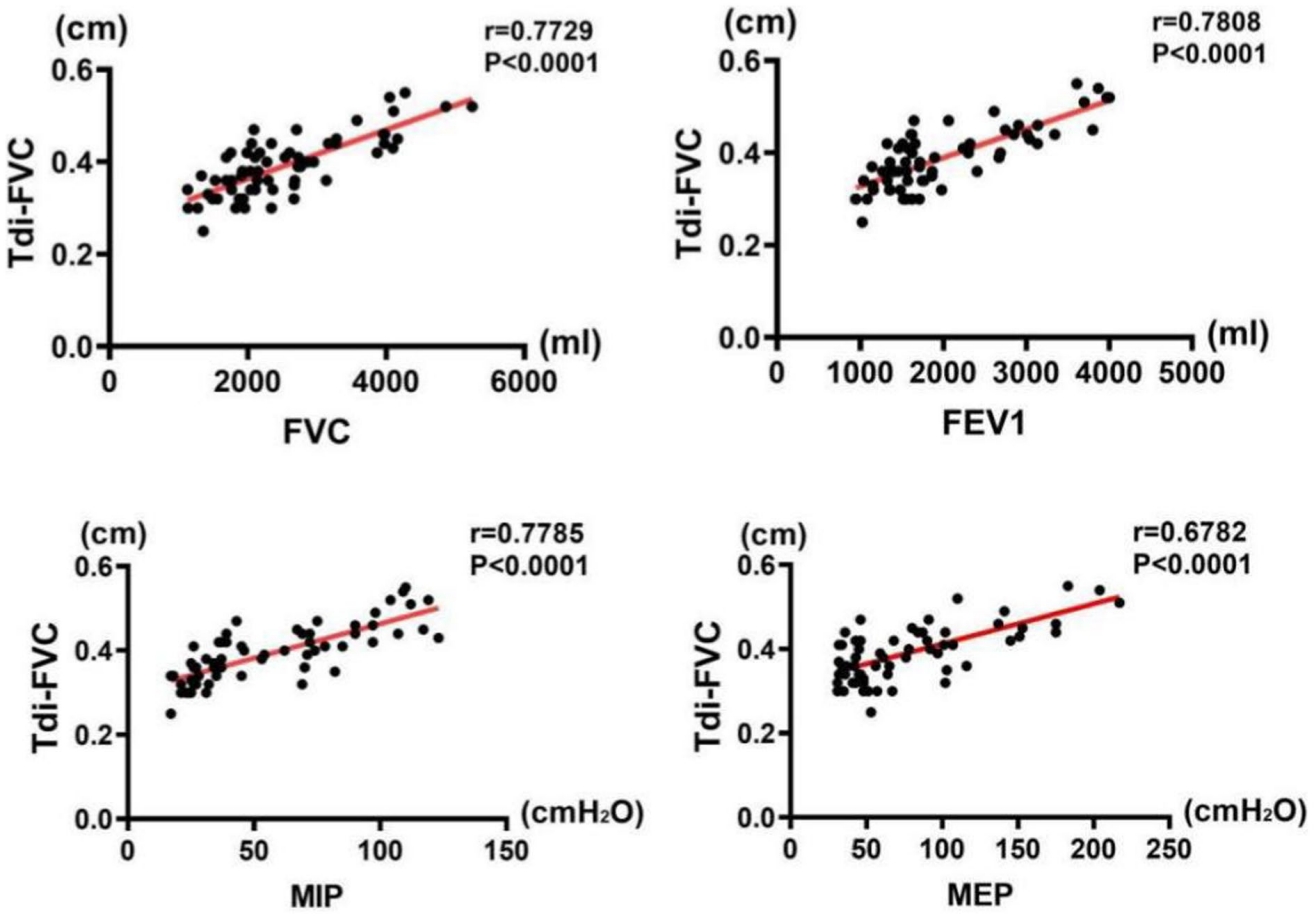
Correlations between pulmonary function indices and TdiFVC.

**Figure 5:**
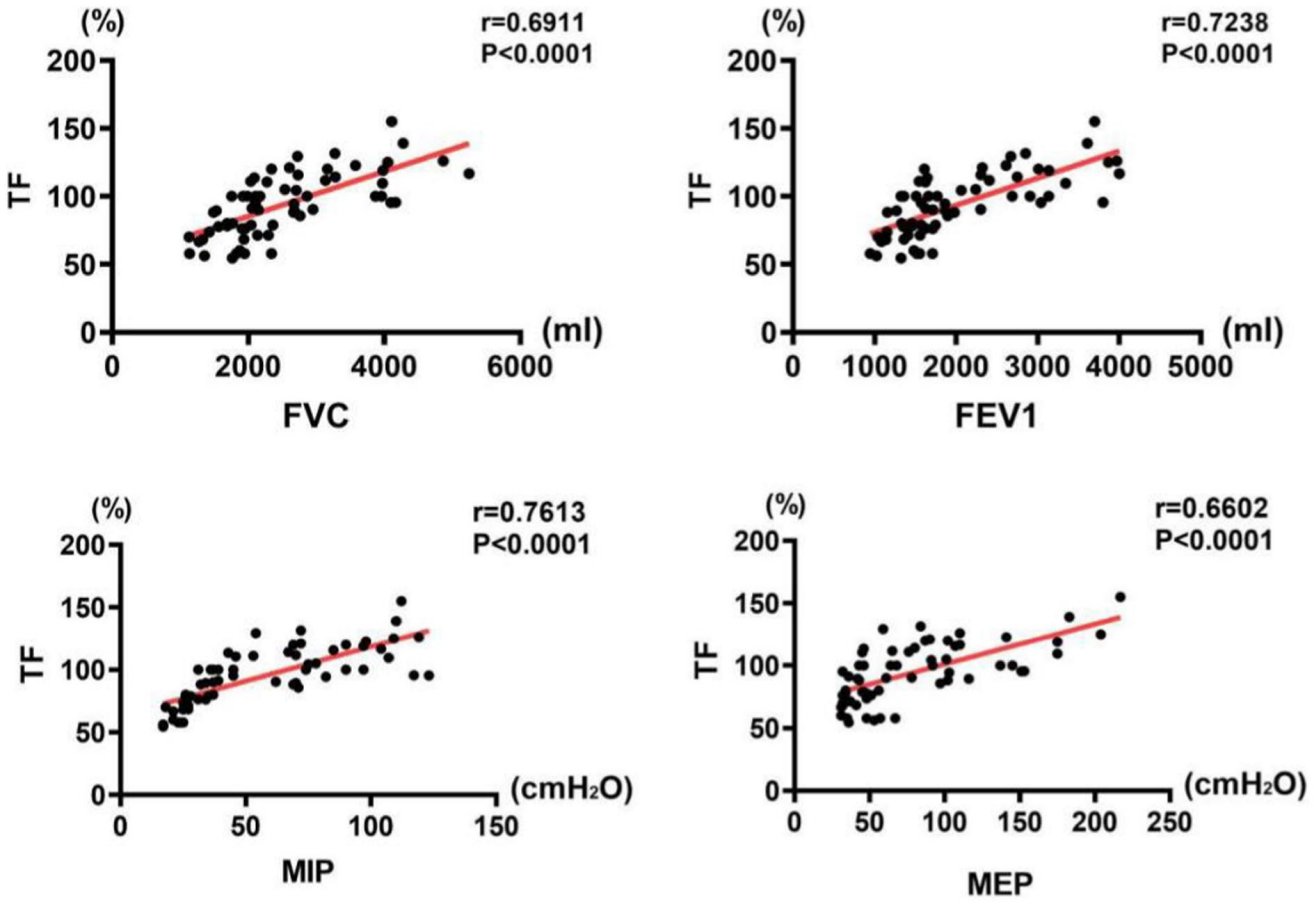
Correlations between pulmonary function indices and TF.

The results of correlation analysis indicated that all pulmonary function indices were moderately correlated with TdiFRC (*p* < 0.001) (Figure 2) and DM (*p* < 0.001) (Figure 3) and strongly correlated with TdiFVC (*p* < 0.001) (Figure 4) and TF (*p* < 0.001) (Figure 5).

### Correlations between pulmonary function indexes and assessment scales in the stroke group

**Figure 6:**
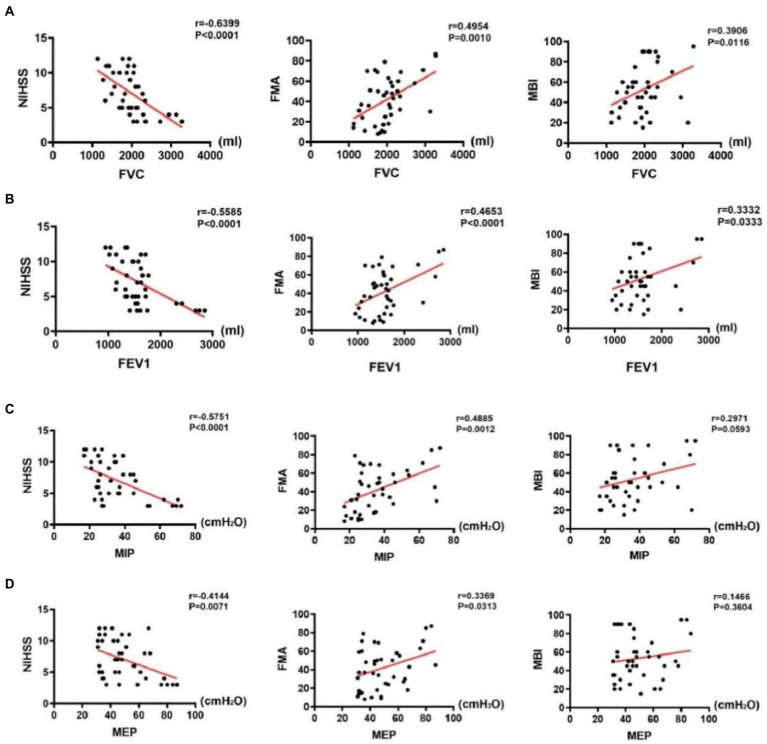
Correlations between pulmonary function indices and assessment scale scores in the stroke group. **(A)** Correlations between FVC and assessment scale scores, **(B)** Correlations between FEV1 and assessment scale scores, **(C)** Correlations between MIP and assessment scale scores, and **(D)** Correlations between MEP and assessment scale scores.

The results of correlation analysis indicated that pulmonary function indices in the stroke group were negatively correlated with NIHSS scores (*p* < 0.001), positively correlated with FMA scores (*p* < 0.001), and weakly correlated (FVC and FEV1, *p* < 0.05) or uncorrelated with MBI scores (MIP and MEP, *p* > 0.05). Details of the analysis are as follows: First, FVC was negatively correlated with NIHSS scores (*r* = −0.6339, *p* < 0.001), positively correlated with FMA scores (*r* = 0.4954, *p* < 0.001), and weakly correlated with MBI scores (*r* = 0.3906, *p* = 0.0116) (Figure 6A). Second, FEV1 was negatively correlated with NIHSS scores (*r* = −0.5585, *p* < 0.001), positively correlated with FMA scores (*r* = 0.4652, *p* < 0.001), and weakly correlated with MBI scores (*r* = 0.3906, *p* = 0.0116) (Figure 6B). Third, MIP was negatively correlated with NIHSS scores (*r* = −0.5751, *p* < 0.001) and positively correlated with FMA scores (*r* = 0.4885, *p* < 0.001); however, it was uncorrelated with MBI scores (*p* > 0.05) (Figure 6C). Fourth, MEP was negatively correlated with NIHSS scores (*r* = −0.4144, *p* < 0.001), positively correlated with FMA scores (*r* = 0.3369, *p* < 0.05), and weakly correlated with MBI scores (*r* = 0.2971, *p* = 0.3604) (Figure 6D).

## Discussion

The results of the present study were consistent with those of previous studies (8, 16, 18) and indicated that the pulmonary function and diaphragmatic ultrasound indices of patients with stroke were decreased compared with those of healthy controls. In previous studies, it has been reported that pulmonary dysfunction in patients with stroke is related to brain injury (19, 20). Changes in the respiratory rate and rhythm as well as weakness of the respiratory muscles may be the direct causes of such injuries (8, 19, 21), and limited thoracic expansion and decreased pulmonary ventilation caused by the weakness of hemiplegic extremities and increased muscle tension may be the indirect causes (22). Furthermore, brain injury directly affects the diaphragmatic muscle on the hemiplegic side, thereby causing muscle weakness, atrophy, and movement reduction (3, 16, 23, 24). Although it has been reported that the nonparalytic diaphragm increases its movement to compensate for the dysfunction of the paralytic diaphragm, the compensation remains insufficient, resulting in a decrease in the vital capacity of patients with stroke (3, 19–21, 25). In addition to the diaphragmatic muscle, other accessory respiratory muscles, such as the sternocleidomastoid, serratus anterior, and intercostal muscles, may be temporarily or permanently deactivated owing to brain damage (5).

In the present study, no significant differences in TdiFRC were found between the two groups; however, there were significant differences in TdiFVC, TF, and DM. This finding may be attributed to the differences in the enrolled patient population. In our study, all patients with stroke were in the convalescent stage, with an average NIHSS score of approximately 9 and an MBI score of 53. Accordingly, their brain injury was moderate, and their motor function was partly recovered (26, 27). Moreover, they could perform some ADLs, such as eating, washing, and dressing, which consumes fewer calories and can be completed in eupnea. However, despite the apparently normal pulmonary function in eupnea, the dysfunction of patients with stroke was evident in deep breathing, as indicated by TdiFVC, DM, and TF, which suggested the decompensation of respiratory function. This could be one of the reasons why the incidence of respiratory complications (e.g., lung infection) is high in these patients and why their disease is more likely to progress to a severe condition after a pulmonary attack. Furthermore, this prompts us to be cautious of pulmonary dysfunction in patients with stroke even though they perform ADLs normally.

In the present study, we found that the mean FVC of patients with stroke was approximately 2 L, which was 56.66% of the mean FVC of the normal age group (Table 1). According to the international guidelines (28), 36 out of 41 patients are diagnosed with restrictive ventilatory disorder. In the present study, the incidence of this disorder was significantly higher in the stroke group than that in the control group (*p* < 0.001). Restrictive ventilatory disorder frequently refers to ventilation dysfunction caused by the limitation of expansion and retraction of the lungs. It is mainly found in the lung tissue; thoracic, cardiac, and mediastinal diseases; and in diaphragmatic paralysis. As previously described, reduced muscle strength of the accessory respiratory muscles, asymmetric activation of the respiratory muscles and movement of the chest wall after stroke (13), abnormal breathing patterns and reduced respiratory flow (6), and a combination of multiple causes limit the patient’s respiratory function, leading to the development of restrictive ventilatory disorders. Moreover, abnormal respiratory patterns and decreased respiratory flow (22) contribute to limited ventilation in patients with stroke. This suggests that respiratory training for patients with stroke should not be limited to the respiratory muscles; rather, general functional training in breathing patterns is crucial. Notably, respiratory rehabilitation has been found to be significant and sufficient. Sutbeyaz (29) reported significant clinical improvements in patients with subacute stroke following inspiratory muscle training, including an increase in lung volume and exercise capacity, the sensation of dyspnea, and quality of life (QOL, 36-Item Short Form Health Survey domains). Furthermore, Dall’Ago (30) reported improvements in patients with cardiac diseases after the training that targeted 30% of MIP compared with the control group with the use of measures for inspiratory strength, functional performance, and QOL.

The diaphragm is the main respiratory muscle as its movement provides approximately two-thirds of the pulmonary capacity. Correlations between pulmonary function and diaphragmatic ultrasound indices have been reported in some studies, including between FEV1, FVC, and TF in patients with osteoporosis, vertebral fracture, and kyphosis (5); between MIP, FVC, TF, and DT in patients with late-onset Pompe disease (6); and between inhalation volume and DM in patients with amyotrophic lateral sclerosis (16). Moreover, other studies of patients with stroke have reported correlations between DT, thickness ratio, and diaphragm excursion and between FVC, FEV1, and peak expiratory flow (8, 9). These findings indicate that a preliminary examination of pulmonary function can be performed through simple diaphragmatic ultrasound detection. Furthermore, we confirmed a positive correlation between pulmonary function and diaphragmatic ultrasound indices (TdiFRC, TdiFVC, DM, and TF) in patients with stroke and healthy controls. Despite the fact that DM is a valuable index, as suggested by other studies (8), we found that TdiFVC is even more significantly correlated with pulmonary function among several indicators of diaphragmatic ultrasound.

It is known that the diaphragm is the main muscle for calm breathing. While contracting during inspiration, it thickens, which causes an increase in the volume of the chest, and more gas rushes into the lungs. However, during forced expiration, the accessory respiratory muscles increase the oxygen demand. Patients with stroke exhibit reduced muscle coordination in the extremities and coordination difficulties in the respiratory muscles. This suggests that the diaphragm plays a more important role in forced expiration, which could be the reason why TdiFVC is more strongly correlated with lung function. In particular, in patients with stroke with seemingly normal pulmonary function under calm breathing, TdiFVC, as an index, can detect underlying diaphragm abnormalities (e.g., insufficient movement under deep breathing) as well as serve as a valuable indicator of pulmonary dysfunction, which is of great significance for pulmonary rehabilitation.

In addition to measuring pulmonary and diaphragm function in patients with stroke, we performed scale assessments. NIHSS is frequently used to evaluate the severity of brain damage, in which higher scores indicate more severe brain damage. MBI is used to assess the ADL of the patient, wherein higher scores indicate better ADL. It has been reported that reduced FVC in patients with stroke is related to worse ADL (31–33). In the present study, we found a moderately negative correlation between pulmonary function and NIHSS scores; however, there was no or weak correlation between pulmonary function and MBI scores (MIP and MEP, *p* > 0.05; FVC and FEV1, *p* < 0.05). This finding may be attributed to the differences in the patient population enrolled. In the present study, patients with stroke had a seemingly normal pulmonary function when performing some ADLs, such as washing, eating, and dressing. Notably, such activities are frequently completed under quiet breathing. Therefore, MBI assessment cannot be used to suspect pulmonary dysfunction in such patients.

This study has some limitations. First, our sample size was comparatively small. Studies with larger sample sizes may provide more positive results. Second, in this study, patients with stroke represented only a population of those with mild-to-moderate neurological impairment after applying our patient screening criteria. Third, as we included patients with a course of stroke ranging from 1 month to 1 year, variations may exist in their assessment. In future, studies may consider narrowing the range of the disease course. Fourth, as the repeatability of ultrasound examination of the left side of the diaphragm is poor owing to limitations in organ anatomy and technology, e.g., the left spleen window is smaller than the right liver window and the measurement on the right side is more feasible than that on the left side (13, 34–36), we included patients with right hemiplegia only for detecting the right side of the diaphragm. Further studies on both sides of the body should be performed considering that the diaphragmatic dysfunction following stroke may be bilateral. Finally, we only examined the diaphragmatic muscles and not the respiratory muscles. Examination of respiratory muscles using other novel techniques, including surface electromyography, optoelectronic plethysmography, and structured light plethysmography, will provide more information about pulmonary dysfunction in patients with stroke.

In conclusion, our results revealed that patients with stroke generally present with pulmonary dysfunction even in the recovery period. Diaphragmatic ultrasound can be used as a simple and effective method for detecting both diaphragmatic function and pulmonary dysfunction in such patients, with TdiFVC being the most effective index.

## Data availability statement

The original contributions presented in the study are included in the article/supplementary material, further inquiries can be directed to the corresponding authors.

## Ethics statement

The studies involving human participants were reviewed and approved by Institutional Ethics Committee of Zhujiang Hospital of Southern Medical University. The patients/participants provided their written informed consent to participate in this study. Written informed consent was obtained from the individual(s) for the publication of any potentially identifiable images or data included in this article.

## Author contributions

All authors listed have made a substantial, direct, and intellectual contribution to the work and approved it for publication.

## Funding

This study was supported by the Medical Science and Technology Research Fund Project of Guangdong Province (no. B2020087).

## Conflict of interest

The authors declare that the research was conducted in the absence of any commercial or financial relationships that could be construed as a potential conflict of interest.

## Publisher’s note

All claims expressed in this article are solely those of the authors and do not necessarily represent those of their affiliated organizations, or those of the publisher, the editors and the reviewers. Any product that may be evaluated in this article, or claim that may be made by its manufacturer, is not guaranteed or endorsed by the publisher.

## References

[1] Katan M Luft A. Global burden of stroke. Semin Neurol. (2018) 38:208–11. doi: 10.1055/s-0038-1649503 29791947

[2] Pelosi P Rocco PR. The lung and the brain: A dangerous cross-talk. Crit Care. (2011) 15:168. doi: 10.1186/cc10259, PMID: 21722336 PMC3219008

[3] Kılıçoğlu MS Yurdakul OV Çelik Y Aydın T. Investigating the correlation between pulmonary function tests and ultrasonographic diaphragm measurements and the effects of respiratory exercises on these parameters in hemiplegic patients. Top Stroke Rehabil. (2022) 29:218–29. doi: 10.1080/10749357.2021.1911748, PMID: 33844946

[4] Schermer TR Jacobs JE Chavannes NH Hartman J Folgering HT Bottema BJ. Validity of spirometric testing in a general practice population of patients with chronic obstructive pulmonary disease (COPD). Thorax. (2003) 58:861–6. doi: 10.1136/thorax.58.10.861, PMID: 14514938 PMC1746497

[5] Ciloglu O Karaali E Gorgulu FF Ekiz T. Diaphragm thickness and stiffness in patients with hyperkyphosis due to osteoporotic vertebral fracture: an ultrasonographic and elastographic study. Pol J Radiol. (2020) 85:575–80. doi: 10.5114/pjr.2020.99751, PMID: 33204371 PMC7654313

[6] Ruggeri P Lo Monaco L Musumeci O Tavilla G Gaeta M Caramori G. Ultrasound assessment of diaphragm function in patients with late-onset Pompe disease. Neurol Sci. (2020) 41:2175–84. doi: 10.1007/s10072-020-04316-6, PMID: 32162165

[7] Heiman-Patterson TD Khazaal O Yu D Sherman ME Kasarskis EJ Jackson CE. Pulmonary function decline in amyotrophic lateral sclerosis. Amyotroph Lateral Scler Frontotemporal Degener. (2021) 22:54–61. doi: 10.1080/21678421.2021.1910713, PMID: 34348540

[8] Jung JH Kim NS. The correlation between diaphragm thickness, diaphragmatic excursion, and pulmonary function in patients with chronic stroke. J Phys Ther Sci. (2017) 29:2176–9. doi: 10.1589/jpts.29.2176, PMID: 29643599 PMC5890225

[9] Kim M Lee K Cho J Lee W. Diaphragm thickness and inspiratory muscle functions in chronic stroke patients. Med Sci Monit. (2017) 23:1247–53. doi: 10.12659/msm.900529, PMID: 28284044 PMC5358861

[10] Huang Q Luo H Yang C Li J Deng Q Liu P. Anatomical prior based vertebra modelling for reappearance of human spines. Neurocomputing. (2022) 500:750–60. doi: 10.1016/j.neucom.2022.05.033

[11] Voyvoda N Yücel C Karatas G Oguzülgen I Oktar S. An evaluation of diaphragmatic movements in hemiplegic patients. Br J Radiol. (2012) 85:411–4. doi: 10.1259/bjr/71968119, PMID: 21712430 PMC3485549

[12] Jung KJ Park JY Hwang DW Kim JH Kim JH. Ultrasonographic diaphragmatic motion analysis and its correlation with pulmonary function in hemiplegic stroke patients. Ann Rehabil Med. (2014) 38:29–37. doi: 10.5535/arm.2014.38.1.29, PMID: 24639923 PMC3953360

[13] Laghi FA Jr Saad M Shaikh H. Ultrasound and non-ultrasound imaging techniques in the assessment of diaphragmatic dysfunction. BMC Pulm Med. (2021) 21:85. doi: 10.1186/s12890-021-01441-6, PMID: 33722215 PMC7958108

[14] Cardenas LZ Santana PV Caruso P Ribeiro de Carvalho CR Pereira de Albuquerque AL. Diaphragmatic ultrasound correlates with inspiratory muscle strength and pulmonary function in healthy subjects. Ultrasound Med Biol. (2018) 44:786–93. doi: 10.1016/j.ultrasmedbio.2017.11.020, PMID: 29373153

[15] Fantini R Mandrioli J Zona S Antenora F Iattoni A Monelli M. Ultrasound assessment of diaphragmatic function in patients with amyotrophic lateral sclerosis. Respirology. (2016) 21:932–8. doi: 10.1111/resp.12759, PMID: 26994409

[16] Cohen E Mier A Heywood P Murphy K Boultbee J Guz A. Diaphragmatic movement in hemiplegic patients measured by ultrasonography. Thorax. (1994) 49:890–5. doi: 10.1136/thx.49.9.890, PMID: 7940429 PMC475186

[17] Scott S Fuld JP Carter R McEntegart M MacFarlane NG. Diaphragm ultrasonography as an alternative to whole-body plethysmography in pulmonary function testing. J Ultrasound Med. (2006) 25:225–32. doi: 10.7863/jum.2006.25.2.225, PMID: 16439786

[18] Annoni JM Ackermann D Kesselring J. Respiratory function in chronic hemiplegia. Int Disabil Stud. (1990) 12:78–80. doi: 10.3109/03790799009166256, PMID: 2254236

[19] Polkey MI Green M Moxham J. Measurement of respiratory muscle strength. Thorax. (1995) 50:1131–5. doi: 10.1136/thx.50.11.1131, PMID: 8553266 PMC475082

[20] Teixeira-Salmela LF Parreira VF Britto RR Brant TC Inácio EP Alcântara TO. Respiratory pressures and thoracoabdominal motion in community-dwelling chronic stroke survivors. Arch Phys Med Rehabil. (2005) 86:1974–8. doi: 10.1016/j.apmr.2005.03.035, PMID: 16213241

[21] Leslie P Drinnan MJ Ford GA Wilson JA. Resting respiration in dysphagic patients following acute stroke. Dysphagia. (2002) 17:208–13. doi: 10.1007/s00455-002-0052-9, PMID: 12140647

[22] Lanini B Bianchi R Romagnoli I Coli C Binazzi B Gigliotti F. Chest wall kinematics in patients with hemiplegia. Am J Respir Crit Care Med. (2003) 168:109–13. doi: 10.1164/rccm.200207-745OC, PMID: 12714347

[23] Park GY Kim SR Kim YW Jo KW Lee EJ Kim YM. Decreased diaphragm excursion in stroke patients with dysphagia as assessed by M-mode sonography. Arch Phys Med Rehabil. (2015) 96:114–21. doi: 10.1016/j.apmr.2014.08.019, PMID: 25234476

[24] de Almeida IC Clementino AC Rocha EH Brandão DC Dornelas de Andrade A. Effects of hemiplegy on pulmonary function and diaphragmatic dome displacement. Respir Physiol Neurobiol. (2011) 178:196–201. doi: 10.1016/j.resp.2011.05.017, PMID: 21679778

[25] Kim J Park JH Yim J. Effects of respiratory muscle and endurance training using an individualized training device on the pulmonary function and exercise capacity in stroke patients. Med Sci Monit. (2014) 20:2543–9. doi: 10.12659/MSM.891112, PMID: 25488849 PMC4266259

[26] Kwah LK Diong J. National Institutes of Health stroke scale (NIHSS). J Physiother. (2014) 60:61. doi: 10.1016/j.jphys.2013.12.012 24856948

[27] Liu F Tsang RC Zhou J Zhou M Zha F Long J. Relationship of Barthel index and its short form with the modified Rankin scale in acute stroke patients. J Stroke Cerebrovasc Dis. (2020) 29:105033. doi: 10.1016/j.jstrokecerebrovasdis.2020.105033 32807445

[28] Pellegrino R Viegi G Brusasco V Crapo RO Burgos F Casaburi R. Interpretative strategies for lung function tests. Eur Respir J. (2005) 26:948–68. doi: 10.1183/09031936.05.00035205 16264058

[29] Sutbeyaz ST Koseoglu F Inan L Coskun O. Respiratory muscle training improves cardiopulmonary function and exercise tolerance in subjects with subacute stroke: A randomized controlled trial. Clin Rehabil. (2010) 24:240–50. doi: 10.1177/0269215509358932, PMID: 20156979

[30] Dall’Ago P Chiappa GR Guths H Stein R Ribeiro JP. Inspiratory muscle training in patients with heart failure and inspiratory muscle weakness: A randomized trial. J Am Coll Cardiol. (2006) 47:757–63. doi: 10.1016/j.jacc.2005.09.052, PMID: 16487841

[31] Yoon J Park J Lee D Roh H. Comparisons of respiratory function and activities of daily living between spinal cord injury and stroke patients and normal elderly people. J Phys Ther Sci. (2012) 24:465–9. doi: 10.1589/jpts.24.465

[32] Lee RN Graydon JE Ross E. Effects of psychological well-being, physical status, and social support on oxygen-dependent COPD patients’ level of functioning. Res Nurs Health. (1991) 14:323–8. doi: 10.1002/nur.4770140503, PMID: 1891618

[33] Ryu SR Shin AY Han JY Choi IS Kim JH Lee SG. The effectiveness of pulmonary rehabilitation program on functional improvement in patients with spinal cord injury. J Korean Acad Rehab Med. (2008) 32:32–7.

[34] Noh DK Koh JH You JS. Inter-and intratester reliability values of ultrasound imaging measurements of diaphragm movement in the thoracic and thoracolumbar curves in adolescent idiopathic scoliosis. Physiother Theor Pract. (2016) 32:139–43. doi: 10.3109/09593985.2015.1091871, PMID: 26863479

[35] Bobbia X Clément A Claret PG Bastide S Alonso S Wagner P. Diaphragmatic excursion measurement in emergency patients with acute dyspnea: Toward a new diagnostic tool? Am J Emerg Med. (2016) 34:1653–7. doi: 10.1016/j.ajem.2016.05.055, PMID: 27251231

[36] Yoon SY Moon HI Kim JS Yi TI Park YG. Comparison between M-mode ultrasonography and fluoroscopy for diaphragm excursion measurement in patients with acquired brain injury. J Ultrasound Med. (2020) 39:535–42. doi: 10.1002/jum.15130, PMID: 31512782

